# The role of Sativex in robotic rehabilitation in individuals with multiple sclerosis: Rationale, study design, and methodology

**DOI:** 10.1097/MD.0000000000008826

**Published:** 2017-11-17

**Authors:** Margherita Russo, Vincenzo Dattola, Anna Lisa Logiudice, Rosella Ciurleo, Edoardo Sessa, Rosaria De Luca, Placido Bramanti, Alessia Bramanti, Antonino Naro, Rocco Salvatore Calabrò

**Affiliations:** IRCCS Centro Neurolesi “Bonino-Pulejo”, Messina, Italy.

**Keywords:** multiple sclerosis, robotic rehabilitation, sativex, spasticity

## Abstract

**Introduction:**

Currently, none of the available multiple sclerosis (MS) disease-modifying medications has been shown to stop or reverse gait disability. Recently, the nabiximols has been tested for the treatment of spasticity and walking impairment in MS. Nabiximols (trade name Sativex) is an oromucosal spray formulation containing 1:1 fixed ratio of delta-9-tetrahydrocannabinol and cannabidiol derived from cloned Cannabis sativa L. plant.

**Method and analysis:**

A single-center, prospective, parallel design, single-blind trial will be conducted at the IRCCS Neurolesi “Bonino-Pulejo” (Italy) involving MS patients affected by spasticity and undergoing a Robotic Rehabilitation training. The aim of the study is to clarify the role of Sativex coupled to a robotic neurehabilitation training in MS patients in improving motor outcomes, by means of clinical, kinematic, and neurophysiological measures. Patients will be randomly divided in 2 groups: one taking only an oral antispastic drug and the other with Sativex in add-on. After 1 month, we will evaluate the response to Sativex (responder patients’ amelioration >20% at MRS score) enrolling into the study the first 20 patients with a good response to Sativex, whereas other 20 no-responder individuals will continue their antispastic drug. All the 40 subjects, were divided into 2 groups (A: Sativex + Lokomat Training, and B: other antispastic+Lokomat Training), will perform a neurorobotic-assisted gait training (each session will last at least 45 minutes, 3 times per week, for a total of 20 sessions). All the patients will undergo a complete physical and neurological examination at baseline, at the end of the robotic training (T1), and 30 days after the end of the neurorehabilitation training (T2).

## 1 Introduction

### 1.1 Background and rationale

Multiple sclerosis (MS) is the second most common cause of neurological disability in adults between 18 and 50 years of age. Gait, coordination, and balance are affected even in people with only mild neurological signs. ^[1]^ Moreover, after early walking problems have been detected, time until complete loss of independent ambulation is predictable, regardless of the relapsing or progressive onset. Spasticity is frequently experienced by people with MS, negatively affecting patient's motor functional outcome, including walking.^[2]^ Currently, none of the available MS disease-modifying medications has been shown to stop or reverse gait disability. Recently, the nabiximols has been tested for the treatment of spasticity and walking impairment in MS. Nabiximols (trade name Sativex) is an oromucosal spray formulation containing 1:1 fixed ratio of delta-9-tetrahydrocannabinol (THC) and cannabidiol (CBD) derived from cloned *Cannabis sativa L.* plant. The main active substance, THC, acts as a partial agonist at human cannabinoid receptors (CB_1_ and CB_2_), and may modulate the effects of excitatory (glutamate [GLU]) and inhibitory (gamma-aminobutyric acid [GABA]) neurotransmitters, leading to muscle relaxation, which in turn is responsible for spasticity improvement.^[3]^ The last 2 decades have seen a remarkable shift of therapeutic approach toward the neurorehabilitation paradigm, considering plasticity as a fundamental property of the human brain. In particular, gait robotic-rehabilitation (RAGT), with or without body weight support (BWS), may offer advantages beyond overground training, in terms of patient safety, reduced fear of falling, more steps practiced, repeatability, and less fatiguing motor paradigms. However, the current data in literature about the efficacy of neurorobotic training in MS are conflicting,^[4]^ although it has been hypothesized that virtual reality may strengthen RAGT thanks to the entrainment of different brain areas involved in motor planning and learning.^[5]^

Nevertheless, the neurorehabilitation plays a key role in preserving the neural-plasticity mechanisms. Cortical reorganization represents the expression of neuronal plastic changes that follow brain tissue damage in MS. Adaptive plasticity is suggested to maintain the expression of pathology at a subclinical level.^[6]^ However, cannabinoid receptors may modulate both excitatory and inhibitory transmission at central synapses, and have been heavily implicated in multiple forms of synaptic plasticity, such as long-term potentiation (LTP) and long-term depression (LTD).^[7]^ Indeed, in a previous study implying transcranial magnetic stimulation (TMS) technique, Koch et al^[8]^ hypothesized that the activation of cannabinoid receptors by Sativex could modulate the balance between LTP and LTD like plasticity by changing the state of cortical excitability. Recently it has been suggested that Sativex may modulate the cortical excitability by changing the activity of the inhibitory GABAergic cortico-cortical synapses.^[3]^

## 2 Objectives

Aim of the study is to clarify the role of Sativex coupled to a robotic neurehabilitation training in improving motor outcomes, by means of clinical, kinematic, and neurophysiological measures in patients with MS affected by spasticity.

## 3 Methods

### 3.1 Trial design

This is a rater-blinded, active-controlled, parallel-group pilot study to assess the efficacy of Sativex associated with robotic-rehabilitation in improving the motor performances of Multiple Sclerosis patients. This study will be conducted at the IRCCS Centro Neurolesi Bonino-Pulejo in Messina, Italy. The study protocol was approved by the Ethical and Research Committee of IRCCS Centro Neurolesi Bonino-Pulejo (13/2016 vers, n 1 23/11/2016). The trial is registered under trial gov. (NCT03186664). The study purpose will be explained to the participants, and patients’ consent will be obtained before the enrollment by the principle investigator.

### 3.2 Eligibility criteria

We will enroll 40 MS patients affected by gait disturbances and moderate-severe spasticity: 20 of them in treatment with Sativex (Group A) considered as responders, and 20 treated only with the most common antispastics used in MS (Group B). Inclusion criteria are: age range 18 to 65 years; diagnosis of definite relapsing-remitting or primary-progressive or secondary-progressive multiple sclerosis; absence of clinical relapses from no gadolinium-enhanced lesions on brain and spinal cord MRI performed at least 6 months before study entry; no mood or sleep disorders with an Hamilton score of ≤17; a moderate to severe spasticity in at least 2 districts of upper and/or lower limbs; absence of clinical or neuroradiological relapses from at least 6 months before study entry; Expanded Disability Status Scale (EDSS) total score between 3.5 and 7.0; no history of psychosis; no presence of pace-maker, aneurysms clips, neurostimulator or brain/subdural electrodes (safety TMS procedure); right handedness, central conduction time in upper limbs of <8 ms; no robotic gait training contraindications.

### 3.3 Exclusion criteria

We consider as exclusion criteria the following: history of psychosis, presence of pace-maker, aneurysms clips, neurostimulator or brain/subdural electrodes (safety TMS procedure), central conduction time in upper limbs of <8 ms; RAGT contraindications.

### 3.4 Prescreening

We evaluated 90 MS patients attending the tertiary MS Center of the IRCCS “Bonino-Pulejo” with a moderate to severe spasticity despite oral antispastic treatment (i.e., Baclofen) and a gait disturbance. We randomly divided the population in 2 groups: one with the same oral therapy and the second with Sativex in add-on. After 1 month, we evaluated the response to Sativex (responder patients’ amelioration >20% at MRS score). We included into the study the first 20 patient responders to Sativex and 20 who continued their ongoing antispastic treatment.

### 3.5 Randomization of participants

The subjects were randomly assigned to one of the two treatment groups, using a simple randomization scheme generated by a software (www.randomization.com). Individual, sequentially numbered index cards with the random assignments were prepared. The index cards were folded, and placed in sealed opaque envelopes

### 3.6 Study population

All the 40 subjects, divided into 2 groups (A: Sativex + Lokomat Training, and B: other antispastics + Lokomat training) will perform a RAGT (each session will last at least 45 minutes, 3 times per week, for a total of 20 sessions).

All the patients will undergo a complete physical and neurological examination, including the evaluation of disability by means of the EDSS, the assessment of spasticity using the Modified Ashworth Scale (MAS), and the numerical rating scale (NRS). To clarify the role of Sativex in improving spasticity gait-related symptoms, we will also administer the following scales: 10 meters walking test (10WT) and Ambulation Index (AI). Quality of life will be evaluated by means of MS quality of life 54 (MSQOL-54). The skilled clinician will be blind to the drug treatment. Moreover, we will evaluate some electrophysiological parameters to test cortical excitability: motor-evoked potentials (MEP) amplitude, short intracortical inhibition (SICI) and facilitation (ICF) from the abductor pollicis brevis muscle (APB) of the most affected side. The same assessment will be applied at baseline, at the end of the RAGT (T1), and 30 days after the end of the neurorehabilitation training (T2).

### 3.7 Study drug

Patients in treatment with Sativex will receive cannabis-based medicine extract presented in a pump action sublingual spray. Sativex is composed of whole cannabis plant extract, containing THC (27 mg/mL) and CBD (25 mg/mL), in ethanol/propylene glycol (50:50) excipient. Each actuation delivers 100 KL of spray, containing THC 2.7 mg and CBD 2.5 mg.

Sativex is indicated as treatment for symptom improvement in adult patients with moderate to severe spasticity due to MS, who have not responded adequately to other antispasticity medication and who demonstrate clinically significant improvement in spasticity-related symptoms during an initial trial of therapy (4 weeks). The drug is intended to be taken with another antispastic medication, and treatment should be started under the supervision of a doctor skilled in MS. The number and frequency of dosing (sprays) with Sativex will vary and the correct dose of Sativex for the individual patient might be reached over a couple of weeks. The most common side effects or adverse effects associated with the first 4 weeks of treatment with Sativex in clinical studies of the drug were dizziness (occurring mostly during the early stages of treatment when the dose is titrated) and fatigue. During the initial Sativex titration period, it is necessary to gradually increase the number of sprays that you take per day. You should leave at least 15 minutes between sprays and should not exceed 12 sprays per day.

### 3.8 Neurorobotic treatment

The neurorobotic treatment will be performed by using the Lokomat (Lokomat, Hocoma, Volketswil, Switzerland) device. The Lokomat is a robotic device, consisting of a powered gait orthosis with integrated computer-controlled linear actuators at each hip and knee joint, a BWS, and a treadmill. Gait pattern and guidance force are individually adjustable to the patient's needs to optimize the functional training in the sagittal, frontal, and transverse planes. Moreover, the new Free-D module allows pelvic rotation and weight shifting with a more physiological deambulation. The system can also evaluate the physiologic stiffness of the patient's hip and knee joints and the isometric force exerted, respectively, for hip and knee extension by the L-STIFF and the L-FORCE software module. The workload will be progressively adjusted based on the improvement of motor performances. Training parameters (weight support, gait speed, guide force) will be individually adapted. During the first Lokomat session, support will be set at 50% of the body weight and will be adapted on observation of the gait. In particular, after 2 weeks, the body weight support will be probably reduced to 30% and in another 2 weeks to 20% of the patient's body weight.

The Lokomat motor guidance system will be first set at 100%, corresponding to a passive walk. The guidance will be reduced as much as possible on observation of the gait pattern. Walking speed will be regulated on observation of gait and will change randomly to simulate normal gait. The locomotor treatment will be administered 3 times per week for 4 weeks. The overall duration of Lokomat therapy, including the time getting in and out, is expected to be 45 minutes, whereas the effective robotic gait training will last about 30 minutes. At the beginning of the treatment, 40% of each patient's body weight will be supported by the harness system.

### 3.9 TMS: recording systems and electrophysiological parameters

MEPs will obtained through magnetic monophasic stimuli delivered through a high-power Magstim200 Stimulator (Magstim, Whitland, Dyfed, UK). The coil will be placed tangentially to the scalp with the handle pointing backwards and laterally, at a 45-degree angle to the sagittal plane, approximately perpendicular to the central sulcus, on the optimal site of the scalp to get the wider MEP amplitude (motor hot-spot), from the APB muscle of the most affected side. The rise time of the magnetic monophasic stimulus will be about 100 μs with a to-zero of about 800 μs. The current flowed in handle direction during the rise-time of the magnetic field, thus with a posterior-anterior direction. We preliminarily will evaluated the resting Motor rMT, defined as the smallest stimulus intensity able to evoke a peak-to-peak MEP of 50 μV in rest APB, in at least 5 of 10 tracks consecutively, and active motor threshold (aMT), defined as the minimum stimulator output that produced MEP of 100 μV more in at least 5 of 10 trials with a constant background contraction of 20% of the maximum integrated electromyography. Then, well applied an intensity of stimulation to obtain an MEP amplitude of ∼0.7 mV. SICI and ICF will be determined according to the paired-pulse method described by Kujirai and co..^[9]^ The intensity of the conditioning stimulus will be set at 80% of aMT. The intensity of the test stimulus (TS) will set to elicit peak-to-peak MEPs amplitude of 0.7 mV. Such intensities will be kept constant throughout the experiment. SICI and ICF will be assessed at an interstimulus interval of 2 and 12 ms, respectively. Mean amplitude of the conditioned MEP will be expressed as percentage of the amplitude of the unconditioned MEP and will be taken as a measure of corticospinal excitability.

Electromyographic activity will be recorded through Ag-AgCl surface electrodes applied to APB using a classic muscle belly-tendon montage. Signals will be amplified and filtered (from 32 Hz to 1 KHz) via a Digitimer D150 Amplifier (Digitimer Ltd., Welwyn Garden City, Herts, UK), and stored using a sampling frequency of 10 KHz on a personal computer for off-line analysis (Signal Software, Cambridge Electronic Design, Cambridge, UK).

### 3.10 Outcome measures

Patients will be clinically evaluated by means of an accurate neurological examination by a blind to treatment physician through validated scales: EDSS, Functional Independence Measure (FIM), MAS, NRS, 10mW, 6 minute walking test (6MW), Hamilton rating scale for Depression (HRSD), and MSQOL 54. Muscular stiffness and strength will be also evaluated by means of instrumental measures furnished by the neurorobotic device itself. Moreover, cortical plasticity will be evaluated by means of TMS methodology. Finally, blood pressure and mean heart rate will be assessed.

### 3.11 Participants Timeline

#### 3.11.1 Pre-screening Visit

Evaluation of inclusion and exclusion criteria. Signature of informed consent.

#### 3.11.2 Visit 1 (baseline)

Medical history with particular regard to antispastic drug intake. FIM, MAs, NRS, 10mW, 6MW, HRSD, and MSQOL 54. Muscular stiffness and strength will be evaluated by means of instrumental measures furnished by the neurorobotic device itself. Neurophysiological measure will be also assessed.

#### 3.11.3 Visit 2 (at the end of rehabilitative program)

FIM, MAs, NRS, 10mW, 6MW, HRSD, and MSQOL 54. Muscular stiffness and strength will be evaluated by means of instrumental measures furnished by the neurorobotic device itself. Neurophysiological measure will be also assessed.

#### 3.11.4 Visit 3 (1 month after the last robotic-rehabilitative session)

FIM, MAs, NRS, 10mW, 6MW, HRSD, and MSQOL 54. Muscular stiffness and strength will be evaluated by means of instrumental measures furnished by the neurorobotic device itself. Neurophysiological measure will be also assessed.

### 3.12 Sample size

On the basis of the pilot samples during study, we calculated a minimal sample size of 36 participants per group in this study, given a power of >80% to detect an interaction in the 2-way repeated measures, an effect size of 0.61, 7 variables, and 4 repeated measurements. Neurophysiological measure will be also assessed.

### 3.13 Statistical analysis

The Wilcoxon signed ranks test on the T0-T1-T2 differences concerning the different clinical outcome measures (EDSS, FIM, MAs, 10mW, 6MW, HSD, MSQOL 54) will be carried out in both groups. The α-level for significance will be set at *P* < 0.05. The Bonferroni correction will be used in multiple comparisons. Data will be expressed as mean or percentage ± standard deviation. The effects of the treatment on RMT, peak-to-peak MEP amplitude, SICI, and ICF will be evaluated in separate repeated-measures analyses of variance (rmANOVA). For each dependent variable, we will compute a 2-way rmANOVA with time (4 levels: T_0_- T_1_-T_2_) and group (2 levels: group A and group B) as within-subject factors. Conditional on a significant F value, post-hoc paired-sample *t* tests were performed to explore the strength of main effects and the patterns of interaction between experimental factors. The Greenhouse-Geisser method will be used if necessary to correct for nonsphericity. All data will be given as means or percent ± standard error. We also will calculate a Fisher Z-transformation concerning the correlations between clinical and electrophysiological parameters.

This is a pilot study. The main aim is to provide evidence which could allow planning of a further confirmatory study. The simple size of 40 patients was determined on the basis of feasibility criteria. Consequently, no formal statistical hypothesis was done

## 4 Discussion

Spasticity is frequently experienced by individuals with MS, negatively affecting patient's functional outcomes.^[2]^ A previous study showed that Sativex is an effective add on option for moderate to severe spasticity in MS patients resistant to existing therapies, as demonstrated by its capability to improve spasticity and been evaluated by Visual Analog Scale and MAS scores.^[10]^ Sativex could be effective thanks to a double effect on intracortical and spinal plasticity modulating the balance between excitatory and inhibitory neurotransmitters.^[3]^ Cannabinoid receptors may modulate both excitatory transmission and inhibitory transmission at central synapses and have been heavily implicated in multiple forms of synaptic plasticity, such LTP and LTD.^[11]^ The RAGT offers many advantages in terms of patient safety, reduced fear of falling, number of steps practiced (intensity of the training), repeatability, and motor paradigm-induced fatigue as compared with the traditional over-ground training and/or treadmill with or without BWS in MS patients.^[12]^ Moreover, Lokomat optimizes sensory inputs that are relevant to step training, repeated practice, and neuroplasticity. A recent study showed that Lokomat training is effective in reducing reflex and intrinsic stiffness (which abnormally increase in spinal cord injury) and improving the abnormal modulation of reflexes over the ankle range-of-motion.^[13]^ There is growing evidence that the association between RAGT and specific antispastic therapy (as Tizanidine) is effective in improving training speed in people with spinal cord injury as compared to Lokomat alone.^[14]^

Keeping in mind all of these factors, a randomized trial was planned among MS patients that experienced spasticity and gait disturbance to investigate the role of Sativex in boosting the Lokomat effect. In relation to the capability of Sativex in modulating cortical plasticity,^[3]^ we can hypothesize that it can improve functional outcome and strengthen RAGT in effectively improving gait, balance.

## 5 Ethics and dissemination

### 5.1 Ethical requirements

The Principal Investigator (PI) will conduct the study according to the current version of Declaration of Helsinki, the Good Clinical Practice guidelines, and the local regulations.

### 5.2 Ethics committee

The PI will obtain ethics committee approval of the protocol, informed consent form, and other required study documents before starting the study. After approval, the informed consent will not be altered without the agreement of the relevant ethics committee. It is the responsibility of the PI to ensure that all aspects of institutional review are conducted in accordance with current governmental regulations. Protocol amendments will be subject to the same requirements as the original protocol. A progress report will be submitted to the ethics committee at required intervals and not less than annually. At the completion or termination of the study, the PI will submit a close-out letter to the ethics committee.

### 5.3 Subject information and consent

A written informed consent will be obtained from all study participants, according to local regulations. The Investigator will inform the subject that participation to the study is voluntary and that refusal will not lead to loss of any benefit or prejudice the relationship with the physician in any way. Before enrolment into the study, each subject will receive a full explanation of the nature and purpose of the study from the Investigator. A clear Information Sheet covering all important aspects in writing will be given to the subject who will read it and have the opportunity to ask any questions whatsoever. The subject will be given adequate time for consideration before he/she is requested to sign the informed consent in duplicate. One of the original copies of the signed informed consent form will be kept by the Investigator.

### 5.4 Subject confidentiality and data protection

Before any testing under this protocol, the subjects will also provide all authorizations required by local law (D.Lgs. 196/2003). In agreement with the GCPs, each subject will be identified by a code in an unequivocal manner, which will be the identifier of the subject for the duration of the study.

### 5.5 Study time table and duation

- Projected first patient in: December 2016
- Projected number of patients: 40 MS patients
- Projected last patient visit: December 2017

12 month-duration
